# Transcriptome Kinetics of Circulating Neutrophils during Human Experimental Endotoxemia

**DOI:** 10.1371/journal.pone.0038255

**Published:** 2012-06-05

**Authors:** Stan de Kleijn, Matthijs Kox, Iziah Edwin Sama, Janesh Pillay, Angela van Diepen, Martijn A. Huijnen, Johannes G. van der Hoeven, Gerben Ferwerda, Peter W. M. Hermans, Peter Pickkers

**Affiliations:** 1 Laboratory of Pediatric Infectious Diseases, Radboud University Nijmegen Medical Centre, Nijmegen, The Netherlands; 2 Intensive Care Medicine, Radboud University Nijmegen Medical Centre, Nijmegen, The Netherlands; 3 Anesthesiology, Radboud University Nijmegen Medical Centre, Nijmegen, The Netherlands; 4 Centre for Molecular and Biomolecular Informatics, Radboud University Nijmegen Medical Centre, Nijmegen, The Netherlands; 5 Department of Respiratory Medicine, University Medical Center Utrecht, Utrecht, The Netherlands; 6 Parasitology, Leiden University Medical Center, Leiden, The Netherlands; University of São Paulo, Brazil

## Abstract

Polymorphonuclear cells (neutrophils) play an important role in the systemic inflammatory response syndrome and the development of sepsis. These cells are essential for the defense against microorganisms, but may also cause tissue damage. Therefore, neutrophil numbers and activity are considered to be tightly regulated. Previous studies have investigated gene transcription during experimental endotoxemia in whole blood and peripheral blood mononuclear cells. However, the gene transcription response of the circulating pool of neutrophils to systemic inflammatory stimulation *in vivo* is currently unclear. We examined neutrophil gene transcription kinetics in healthy human subjects (n = 4) administered a single dose of endotoxin (LPS, 2 ng/kg iv). In addition, freshly isolated neutrophils were stimulated *ex vivo* with LPS, TNFα, G-CSF and GM-CSF to identify stimulus-specific gene transcription responses. Whole transcriptome microarray analysis of circulating neutrophils at 2, 4 and 6 hours after LPS infusion revealed activation of inflammatory networks which are involved in signaling of TNFα and IL-1α and IL-1β. The transcriptome profile of inflammatory activated neutrophils *in vivo* reflects extended survival and regulation of inflammatory responses. These changes in neutrophil transcriptome suggest a combination of early activation of circulating neutrophils by TNFα and G-CSF and a mobilization of young neutrophils from the bone marrow.

## Introduction

Sepsis is the most frequent cause of death at non-cardiac intensive care units [1], [2]. It is characterized by a systemic inflammatory response, and associated with marked cardiovascular changes, capillary leakage, tissue damage and multiple organ failure. The most common form of sepsis is caused by bacterial infection, and involves a severe host immune response. The inflammatory responses induced by bacteria are mediated by the expression of pathogen-associated molecular patterns (PAMPs) which are recognized by the host cells via pattern recognition receptors (PRRs) such as Toll-like receptors (TLRs) [3]. For example, stimulation of TLR4 by lipopolysaccharide (LPS) of Gram-negative bacteria results in activation of inflammatory signaling pathways and induction of cytokine secretion by endothelium and circulating leukocytes [4]. In blood, systemic inflammation is classically reflected by a change in leukocyte count with most prominent an increase in neutrophils. During the course of systemic inflammation, neutrophil numbers remain elevated [5]. Besides their anti-microbial activity, neutrophils also exert regulatory functions during inflammation by secretion of their granular content and direct cell-cell interaction with mononuclear cells and endothelium [6], [7]. It has been shown that the pool of circulating neutrophils during systemic inflammation is reflected by a diversity in neutrophil phenotype [8].

The human endotoxemia model is a valuable tool to study early inflammatory mechanisms [9]. Transcriptome analysis of this *in vivo* response to LPS has been shown to reflect the trancriptome profiles of septic or injured patients[10]–[12]. These studies focused on whole blood and PBMC analysis, showing an early acute response followed by a recovery phase in gene expression. However, up till now, it is unclear what part of this acute systemic inflammatory regulation can be attributed to circulating neutrophils. In the current study, we present for the first time the transcriptional response of circulating neutrophils to LPS administration *in vivo*. We use novel analysis methods based on physical protein interaction to control for interaction enrichment bias. Hereby, we aim to identify the role of circulating neutrophils in controlling systemic inflammation by evaluation of their gene expression kinetics and immunomodulatory properties during early inflammatory responses, increased granulopoiesis and neutrophilia.

## Results

### Clinical and Inflammatory Parameters Induced by LPS Infusion in Healthy Volunteers

**Figure 1:**
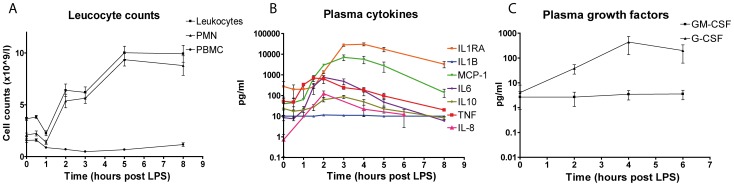
Clinical parameters in time after LPS infusion. A, Leukocyte count with total leukocytes, Polymorphonuclear cell fraction and mononuclear cell fraction. Error bars represent SEM (N = 4). B, Plasma cytokines at different time points measured by luminex or ELISA (IL8). Error bars represent SEM (N = 4). C, Plasma GM-CSF and G-CSF measured by cytometric bead array. Error bars represent SEM (N = 4).

Infusion of LPS in healthy volunteers induced clinical symptoms corresponding with a systemic inflammatory response (increased heart rate, increased body temperature) (data not shown). Total leukocyte counts increased 1–2 hours after infusion of LPS and remained elevated during the whole experiment (12 hours) (Figure 1A). Leukocyte counts showed a depletion of monocytes and lymphocytes 1 hour after LPS infusion with gradual recovery afterwards. In contrast, the neutrophil fraction after a short drop in numbers (50%), increased 1 hour after LPS infusion and remained elevated during the entire experiment (Figure 1A). Plasma levels of pro-inflammatory cytokines TNFα and IL-6 levels were maximal 1.5 hours after infusion of LPS and returned to baseline after 6 hours, whereas circulating IL-1β remained below the detection limit. The anti-inflammatory cytokines IL-1RA and IL-10 reached maximal plasma levels 3 hours after LPS infusion. The chemokines IL-8 and MCP-1 peaked at 2 hours and 3 hours after LPS infusion, respectively (Figure 1B). The colony stimulating factor G-CSF peaked at 4 hours after LPS infusion, whereas plasma levels GM-CSF remained below detection limit (Figure 1C).

### Microarray Transcriptome Analysis of Circulating Neutrophils after LPS infusion Reveals a Time Specific Expression Pattern

To investigate the *in vivo* neutrophil transcription response after LPS infusion, a total of 17393 core genes was analyzed on microarray. Principal component analysis of expression in time indicates a separation of time t = 2 hours from time t = 4 hours and t = 6 hours for all four subjects (data not shown). From the core gene set, 2248 genes (≈13%) were differentially expressed relative to time t = 0 hours at a fold change threshold of 2 (Table S1). Out of these genes, 233 genes were consistently upregulated and 307 genes were consistently downregulated at all 3 investigated time points following LPS infusion. Gene ontology analysis indicates overrepresentation of processes involved in the inflammatory response in the up-regulated genes and lymphocyte activation in the down-regulated genes (Figure S1).

**Figure 2:**
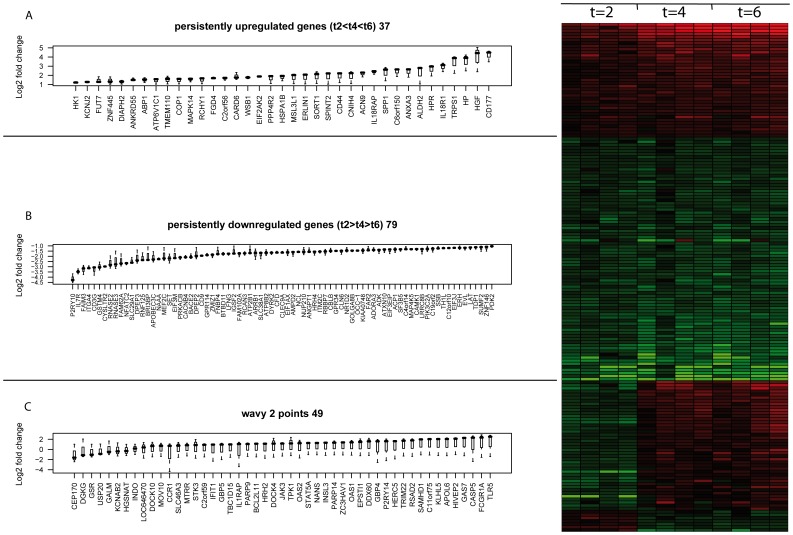
Persistent changes in gene expression. A Genes which are persistently upregulated in time, B Genes which are persistently downregulated in time, C Genes which are regulated in opposite direction between t = 2 h and t = 4 h/t = 6 h after LPS infusion (wavy genes). D, Heat map with fold change of genes relative to t = 0. Green represents downregulation and red upregulation.

Out of the 2248 differentially expressed genes, 37 were increasingly upregulated and 79 genes were increasingly downregulated over time. Meanwhile, 49 genes showed a ‘wavy’ behavior in time, being either first upregulated at t = 2 hours and downregulated at t = 4 hours and t = 6 hours or vice versa. This was consistent for all four subjects tested (Figure 2).

### Validation of Microarray by q-PCR

**Figure 3:**
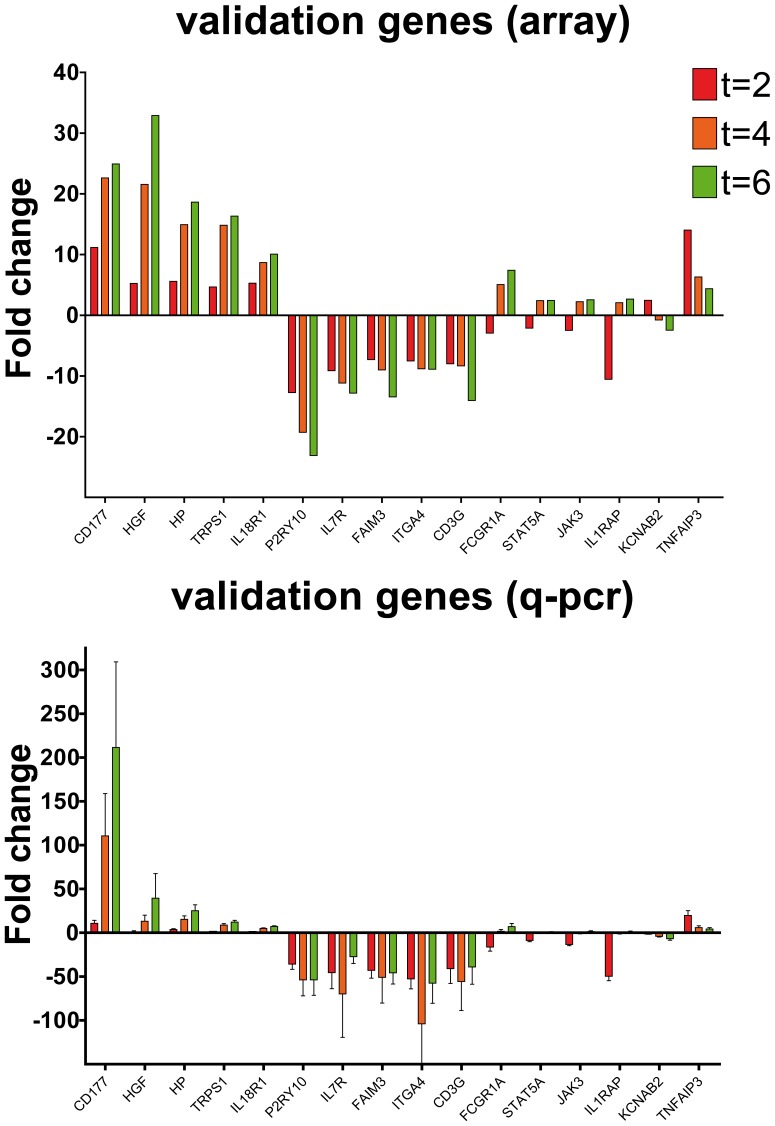
Validation of array results by taqman gene expression assay q-PCR. A, Gene expression in time relative to t = 0 for 16 target genes Geometric mean of 4 individuals. B, corresponding q-PCR results for same geneset in time relative to t = 0 h.

The top 5 persistently upregulated and the top 5 persistently downregulated genes were selected for q-PCR validation of the microarray, as well as 5 relevant inflammatory genes with wavy behavior (Table 1). Q-PCR gene expression analysis was similar with the expression data from the microarray (Figure 3). Strongly downregulated IL7R and CD3G confirm minor PBMC contamination in isolated neutrophil fractions.

**Table 1:** Selection of top 5 persistently upregulated, persistenly downregulated and relevant wavy genes for q-PCR validation with fold changes on microarray.

	Gene symbol	t = 3	t = 5	t = 7	Description					
persistently upregulated	CD177	11.16	22.63	24.93	CD177 antigen					
	HGF	5.24	21.56	32.90	Hepatocyte growth factor					
	HP	5.58	14.93	18.64	Haptoglobin					
	TRPS1	4.66	14.83	16.34	Trichorhinophalangeal syndrome 1					
	IL18R1	5.28	8.69	10.06	Interleukin 18 receptor 1					
persistently downregulated	P2RY10	−12.73	−19.29	−23.10	Purinergic receptor P2Y, G-protein coupled, 10					
	IL7R	−9.13	−11.16	−12.82	Interleukin 7 receptor					
	FAIM3	−7.31	−9.00	−13.45	Fas apoptotic inhibitory molecule 3					
	ITGA4	−7.52	−8.82	−8.88	Integrin alpha 4 (antigen CD49D, alpha subunit of VLA-4 receptor)					
	CD3G	−8.00	−8.34	−14.03	CD3G molecule, gamma (CD3-TCR complex)					
Wavy behavior	FCGR1A	−2.95	5.06	7.41	Fc fragment of IgG, high affinity 1a, receptor (CD64)					
	STAT5A	−2.11	2.41	2.43	signal transducer and activator of transcription 5A					
	JAK3	−2.48	2.23	2.55	Janus Kinase 3					
	IL1RAP	−10.56	2.08	2.66	Interleukin 1 Receptor accessory protein					
	KCNAB2	2.45	−0.77	−2.46	potassium voltage gated channel, shaker related subfamily, beta member					

### Consistently Upregulated and Downregulated Genes have a High Number of Protein-Protein Interactions among them

**Figure 4:**
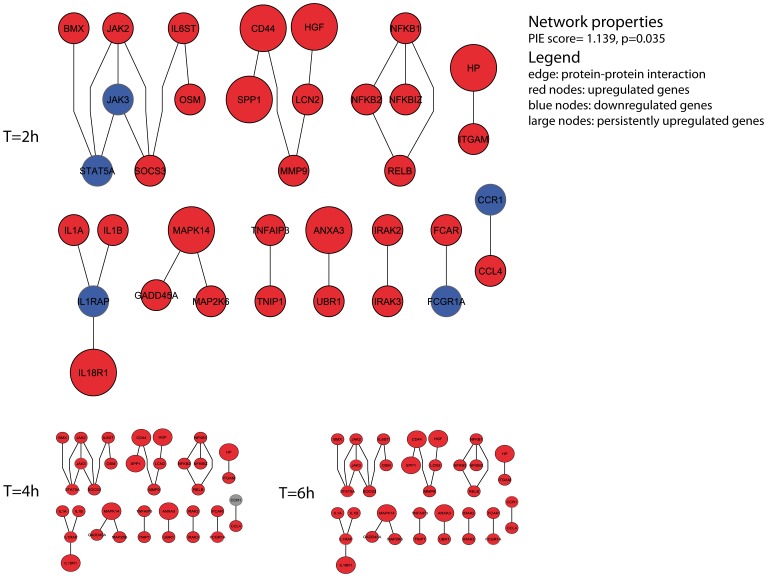
Functional networks of persistent changes in gene expression. Cohesive network based on 233 upregulated genes. ‘Wavy’ genes are marked blue and persistent upregulated genes are represented by large nodes.

The higher the number of protein-protein interactions within a set of proteins, the more functionally cohesive the set is expected to be. The overrepresentation of interactions among a set of proteins, relative to a random set of proteins with the same number of interactions among all known protein-protein interactions, can be expressed with a Physical Interaction Enrichment (PIE) score [13]. The PIE score for randomly chosen genes is expected to be 1.0, P-values are obtained by randomly sampling sets of genes. To identify relevant networks involved in the neutrophil kinetics after LPS infusion, we analyzed the genes that were consistently upregulated (233) or downregulated (307). Both the consistently upregulated and the consistently downregulated genes show a high number of protein-protein interactions among them (PIE score 1.139, p = 0.053 for the upregulated genes and PIE score 1.274, p = 0.002 for the downregulated genes), indicating that they are functionally cohesive. The protein-protein interaction network of up-regulated genes contains interacting proteins involved in signaling of TNFα and IL-1, regulators of transcription (NFκB family), apoptosis and ligand-receptor interaction (Figure 4). IL-1A was strongly upregulated (68 fold increase) at 2 hours after LPS (Table S1).

### TNF-signaling, Inflammatory Networks and Apoptosis Networks are Significantly Influenced by LPS Infusion

**Figure 5:**
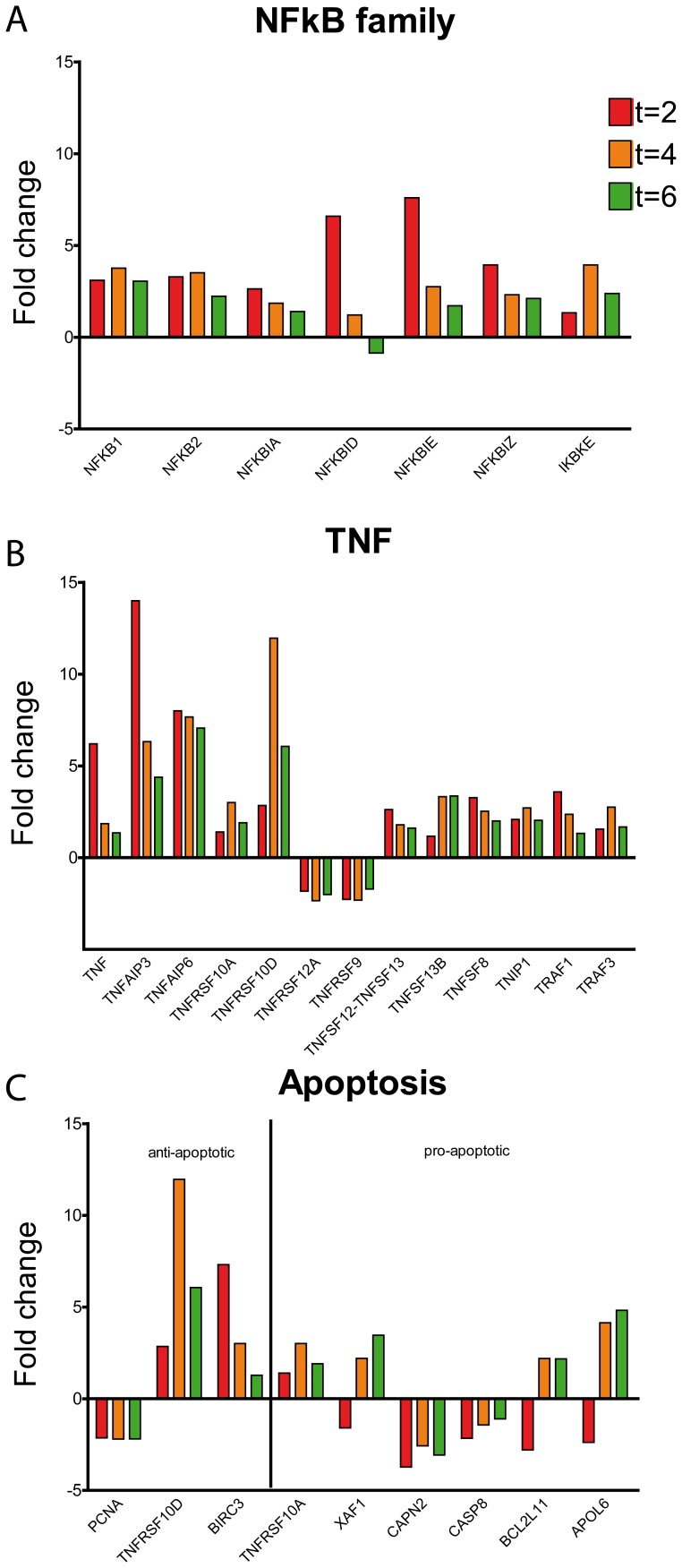
Kinetic behavior of inflammatory genes. A Genes of the NFκB family. Fold change in time relative to t = 0 h. B TNF related genes and genes from TNF receptor family. Fold change in time relative to t = 0. C Apoptosis mediating genes. Fold change in time relative to t = 0 h.

Most of the upregulated genes that are involved in inflammatory pathways and genes of the TNFα signaling pathway were affected early after LPS infusion. In general, transcription factor expression for inflammatory genes was induced by upregulation of NFκB-family genes NFKB1, NFKB2, NFKBIA, NFKBID and NFKBIE(Figure 5A).

Since TNFα plasma levels increased early following LPS infusion in parallel with circulating neutrophils, we investigated the activation of neutrophils by TNFα. TNFα inducible proteins 3 and 6 were strongly upregulated at all time points with peaks at t = 2 hours. TNFAIP3 was because of its important role in TNFα signaling also validated by q-PCR (Figure 3). Furthermore, TRAF genes were induced and TNF receptor gene expression was differentially regulated (Figure 5B). TNFα gene expression was upregulated at t = 2 hours, but not at t = 4 hours and t = 6 hours. These data may suggest that circulating neutrophils after LPS infusion are activated by circulating TNFα.

Apoptosis of neutrophils is inhibited by G-CSF and interestingly, apoptosis of circulating neutrophils is decreased during sepsis [14]. Early after LPS infusion in healthy volunteers plasma levels of G-CSF increased (Figure 1C). Apoptosis pathway genes were differentially regulated, with among them a pronounced upregulation of anti-apoptotic genes BIRC3 and TNFRSF10D (Figure 5C).

### Specific Ex vivo Stimulation of Neutrophils Results in Stimulus Specific Gene Expression Patterns

**Figure 6:**
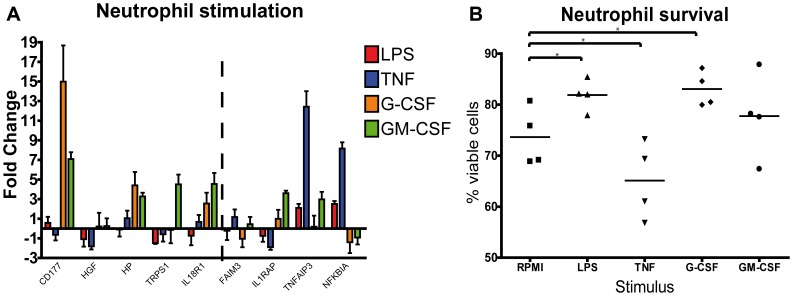
Ex vivo neutrophil stimulation. A Gene expression in *in vitro* stimulated neutrophils. Cells were stimulated with 10 ng LPS, 10 ng rTNFα, 50 ng rG-CSF or 50 ng rGM-CSF. At t = 2 h after stimulation RNA was isolated and q-pcr was performed with taqman probes for specific genes. Fold change relative to unstimulated. Error bars represent SEM (N = 4). B Survival after stimulation with 10 ng LPS, 10 ng rTNFα, 50 ng rG-CSF or 50 ng rGM-CSF. Cell viability was determined in ≥1×10^5^ cells with Annexin V apoptosis detection kit at 7 hours after stimulation. Dots represent the percentage of viable (Annexin V negative and 7 AAD negative) cells. N = 4 *p<0.05.

The induction of the increasingly upregulated genes and TNF/anti-apoptosis pathways is initiated by multiple ligands. We investigated the effect of direct activation by specific ligands on neutrophil gene transcription. Freshly isolated neutrophils were stimulated *ex vivo* with LPS, rTNFα, rG-CSF and rGM-CSF. Figure 6A shows neutrophil gene expression at 2 hours after stimulation. The neutrophil specific protein CD177 was mainly induced by G-CSF (15 fold increase) and TNFα induced TNFAIP3 expression (12 fold increase) as well as NFKBIA (8 fold increase). Other investigated genes were not mediated by these specific ligands.

In addition, the *ex vivo* survival of neutrophils was assessed. neutrophils incubated with LPS and G-CSF for 7 hours showed 10% more viable cells compared with RPMI control, and GM-CSF stimulated cells (Figure 6B). TNFα stimulated neutrophils exhibited 10% lower viable cells compared with RPMI. These results confirm that the activation, reflected by gene-expression patterns, of circulating neutrophils can be partially induced by the early secreted cytokines, growth factors, and direct stimulation with LPS.

## Discussion

In our study, we characterized the transcriptome profiles of circulating neutrophils, in the human experimental endotoxemia model. We showed an abundant upregulation of inflammatory pathways and regulation of apoptosis pathways involving G-CSF, TNFα and IL-1α and IL-1β [15].

The microarray analysis revealed direct neutrophil responses to a diverse pool of activators present in high concentrations in the circulation after LPS infusion. Clear distinction can be made between early (2 hours after LPS) and late (4 hours and 6 hours after LPS) response transcriptome profiles with highest activation at 2 hours after LPS. In contrast, Calvano et al. showed a time dependent activation of inflammatory pathways with peak activation at 4 hours in whole blood [10]. It is unclear what causes these differences since the total circulating leukocyte population after activation exists mainly out of neutrophils. Interestingly gene transcription at 2 hours is similar with our current findings and reflected by e.g. early elevated expression of IL1A, IL1B TNF, TNFAIP3, PTX3 and different chemokines like CCL20 and CXCL10. This suggests that the by Calvano et al. reported change in whole blood inflammatory gene transcription at 2 hours after LPS administration can be mainly attributed to circulating neutrophils. Furthermore, others have shown previously in this model of systemic inflammation that after LPS administration a population of CD16^dim^ neutrophils with a banded nuclear morphology appears in the circulation, most likely released from the bone marrow [8]. This population comprises up to 40% of the total amount of circulating neutrophils at 6 hours after LPS administration. In this respect, it is very likely that changes in gene expression at 4 hours and 6 hours after LPS are partially due to an increase of these young CD16^dim^ neutrophils in the circulation. The influence of these CD16^dim^ neutrophils could be even higher since these cells have a lower density and the recovery from our density centrifugation method may be less.

The novel analysis method based on physical protein interaction, enabled us to identify networks of upregulated genes linked with inflammatory activation and apoptosis of neutrophils without interaction enrichment bias. This upregulated functional network showed a strong prediction of physical protein interaction as indicated by its high cohesiveness [13]. By restricting to the protein interaction network with high cohesiveness, we identified potential relevant protein interactions for neutrophils in early inflammatory response. The current analysis revealed a network of eleven different protein interactions that may account for the inflammatory regulation of circulating neutrophils. Since multiple upregulated genes such as Matrix Metalloproteinase 9 (MMP9), Lipocalin 2(LCN2), Hepatocyte growth factor (HGF) and Haptoglobin (HP), are known as granule proteins, the backbone of upregulated genes we found suggests a change in granule content following activation by pro-inflammatory factors. Interestingly, the expression of these proteins is increased in bone marrow neutrophils and their precursors[16]–[18]. Since HGF and HP expression are poorly upregulated by specific *ex vivo* stimulation (Figure 6), it is plausible that the observed increases in expression are partially represented by release of bone marrow neutrophils to the circulation. This is supported by our findings that upregulation of the neutrophil specific marker CD177, highly expressed on bone marrow neutrophils, is even higher at 4 hours and 6 hours after LPS compared with 2 hours after infusion. In contrast with this, we did show G-CSF induced upregulation of CD177 expression *ex vivo*. This is confirmed by Passamonti et al., who showed that neutrophils isolated from the bone marrow, or from peripheral blood following G-CSF administration showed both increased CD177 expression compared with circulating granulocytes in steady state conditions [19]. Taken together, this implies that direct stimulation of circulating neutrophils and influx of bone marrow neutrophils contribute both to changes in gene expression kinetics.

During systemic inflammation, pro-inflammatory pathways (LPS,TNFα) and pro-survival routes (G-CSF, GM-CSF) can cause neutrophilia by induction of neutrophil release from the bone marrow [20], [21]. In addition to release from the bone marrow, delayed neutrophil apoptosis may also contribute to high circulating neutrophil numbers. During sepsis, a delayed apoptosis can result in neutrophilia and subsequent tissue damage [22]. It has been demonstrated that already under steady state conditions, *in vivo* neutrophil lifespan is about 5.4 days [23]. We showed under early inflammatory conditions that apoptosis pathways are regulated on the gene expression level. The gene expression profiles indicated delayed neutrophil apoptosis by either early induced expression of anti-apoptotic routes like the TNFα pathway but also G-CSF driven inhibition of pro-apoptotic routes via Calpain and XAF1 [24]. In line, *ex vivo* stimulation of neutrophils with G-CSF reduced apoptosis. In contrast, stimulation with TNFα even increased apoptotic cell death. This implies that although the TNFα survival pathway was upregulated, TNFα has no anti-apoptotic effect early after stimulation.

As described recently, IL-1β inhibits IKKβ-NF-κB and thereby contributes to neutrophilia [25]. The upregulated protein interaction network after LPS infusion suggests increased IL-1 signaling although circulating IL-1β was not detectable. Since IL-1α expression was strongly increased in neutrophils at 2 hours after LPS infusion (Table S1), it may indicate an important role of IL-1α in the observed neutrophilia and activated IL-1 signaling pathway. This supports our hypothesis that the observed neutrophilia, at least in part is caused by cytokine and growth factor increased survival of circulating neutrophils. In neutrophils, the role of IL-1α in the induction of survival might be superior compared to TNFα. The contribution of IL-1α in the mediation of survival in neutrophilia should therefore be further investigated.

We hereby directly demonstrated early pro-inflammatory activation of neutrophils reflected by induced pathway expression. Since LPS is cleared from the circulation very rapidly, high numbers of circulating inflammatory cytokines, released by a.o tissue macrophages [26] and endothelium, are likely to account for the major part of this activation of neutrophils. TLR pathways are induced in macrophages and endothelial cells directly after infusion of LPS [27], [28]. We found no persistent upregulation of these pathways in circulating neutrophils. We did show that TNF signaling routes were activated and NFκB family genes were induced These genes comprised mainly the NFκB inhibitory complex like NFKBIA, NFKBID, NFKBIE and NFKBIZ. This is in line with neutrophil transcriptome data in sepsis patients indicating suppression of neutrophils during sepsis [29]. Our data suggests that these regulatory mechanisms are also changed during controlled human endotoxemia. The ability of LPS to directly regulate neutrophil activity is mainly dependent on the TLR expression on neutrophils. Interestingly LPS was able to slightly increase neutrophil survival *ex vivo*. Since neutrophils do express TLR4 [30], it is plausible that LPS can activate these pathways, however this activation is not reflected on the most persistently upregulated genes.

This is the first full genome gene transcription analysis that has been performed in circulating neutrophils during human experimental endotoxemia. We herewith include the complex environment of the circulation after systemic activation. By combining this analysis with specific *ex vivo* neutrophil stimulation we underlined the complexity of neutrophil kinetics during early systemic inflammation. We show that gene transcription profiles of inflammatory activated neutrophils *in vivo* reflects extended survival and regulation of inflammatory responses. These changes in neutrophil transcriptome suggest a combination of early activation of circulating neutrophils and a mobilization of young neutrophils from the bone marrow.

## Methods

### Subjects

Neutrophil gene expression was studied in 4 healthy male volunteers who participated in a human endotoxemia trial (Clinical Trial Register number NCT00783068, placebo group). The study protocol was approved by the Ethics Committee of the Radboud University Nijmegen Medical Centre and complies with the Declaration of Helsinki including current revisions and the Good Clinical Practice guidelines. Written informed consent was obtained from all study participants. Physical examinations, electrocardiography, and routine laboratory studies on all the volunteers before the start of the experiment showed normal results. Volunteers were not taking any prescription medications, and tested negative for hepatitis B surface antigen and human immunodeficiency virus infection.

### Human Endotoxemia Model

Subjects refrained from food 12 hours before the start of the experiment, and caffeine or alcohol containing substances 24 hours before the start of the experiment. The experiments were performed according to a strict clinical protocol as described previously [26]. U.S. Reference *E. coli* endotoxin (*Escherichia coli* O:113, Clinical Center Reference Endotoxin, National Institute of Health (NIH), Bethesda, MD) was used. Ec-5 endotoxin, supplied as a lyophilized powder, was reconstituted in 5 ml saline 0.9% for injection and vortex-mixed for at least 10 minutes after reconstitution. The endotoxin solution was administered as an intravenous bolus injection at a dose of 2 ng/kg of body weight.

### Plasma Cytokine and Growth Factor Measurements

During human endotoxemia experiments, EDTA anticoagulated blood was collected from the arterial line and immediately centrifuged at 2000 g for 10 minutes at 4°C to obtain plasma. Concentrations of IL-1β, TNF-α, IL-6, IL-10, IL-1RA and MCP-1 in plasma and whole blood stimulation supernatants were measured using a simultaneous Luminex Assay according to the manufacturer’s instructions (Bio-plex cytokine assay, BioRad, Hercules, CA, USA). IL-8 was measured in plasma by ELISA (Pelipair, Sanquin, Amsterdam, the Netherlands) following manufacturer’s protocol and plasma G-CSF and GM-CSF concentrations were measured by cytrometric bead array (BD, Franklin Lakes, USA) on the FACScalibur flow cytometer.

### Neutrophil Isolation

Blood samples were drawn at 0, 2, 4 and 6 hours after LPS administration in 10 ml sodium heparin blood tubes (BD, Franklin Lakes, USA). Total leukocytes were determined by Tuerk’s solution (Merck staining, Darmstadt, Germany). Blood was diluted 1∶1 with PBS and placed on lymphoprep (Axis shield, Dundee, UK) and centrifuged. The 1∶1 diluted blood plasma was stored at −80°C until further use. The lowest compartment containing the polymorphonuclear cell fraction was taken containing neutrophils (purity >95%) mostly contaminated by eosinophils and minor PBMCs. Shock buffer containing 0.155 M NH_4_Cl, 0.0001 M Na_2_EDTA and 0.01 M KHCO_3_ was used to lyse the red blood cells. After washing, the granulocytes were counted on a hemocytometer and cell viability (>99%) was determined using 0.4% trypan blue solution. Granulocytes were suspended at a concentration of 10*10^6^ cells/ml in Qiagen RLT buffer (Qiagen, Venlo, the Netherlands) containing 1% B-mercaptoethanol and stored at −80°C until RNA isolation For *ex vivo* neutrophil stimulation, after obtaining written informed consent, EDTA coagulated blood was drawn from healthy volunteers (N = 4). Neutrophils were isolated according to the same protocol. After isolation and washing, cells were allowed to recover for 30 minutes at 37°C.

### RNA Isolation and Microarray Analysis

RNA was isolated by Qiagen RNAeasy RNA isolation kit according to the manufacturer’s protocol. DNA contamination was removed by on column DNase treatment (Qiagen, Venlo, the Netherlands). Total RNA yield was determined on the nanodrop ND-1000 (Isogen life sciences, and Total RNA quality was assessed at Agilent 2100 bioanalyzer with RNA 6000 Nano chips (Agilent, Santa Clara, USA). Neutrophil gene expression was measured on Affymetrix Human ST 1.0 exon arrays. RNA material was first amplified, transformed to cDNA and labeled using ambion WT expression kit and the affymetrix terminal labeling kit (Ambion, Life Technologies, Carlsbad, USA). Labeled cDNA was then hybridized for 17 hours to a Human ST 1.0 exon array, washed and stained according to manufacturers’ instructions and scanned on a Genechip ® scanner 3000 (Affymetrix, Santa Clara, USA). Microarray data has been uploaded to the Gene expression omnibus (GEO) with accessionnumber GSE35590.

### Data Analysis

*Affymetrix*® CEL-files from microarray scans were used for quality control and first Robust Multiarray Averaging (RMA) analysis for normalization was performed with Partek® genomics suite™. In order to assess the extent of gene expression fold changes after LPS infusion in a subject, the normalized log2 intensity value at time 0 was subtracted from those of 2,4 and 6 h, to yield the relative expression fold change per probeset identity. The mean fold changes of all probeset identities pertaining to a gene were used to represent the effective fold change of the gene for that infused subject. The mean fold change for a gene in all 4 subjects was finally used as its overall fold change of perturbation in expression after LPS infusion. Genes that were perturbed at least 2 fold relative to time 0 of LPS infusion were used for subsequent analyses. That is, a gene is considered up-regulated in expression if its log2 fold change value is greater than 1; or down regulated if its log2 fold change value is less than −1. At this fold change threshold, we queried for sets of genes that are perturbed in expression at any time point (i.e. major overall perturbed gene set); at each time point; at all time points; only at certain time points; those that are increasingly up-regulated or down-regulated in the course of infusion; and finally those that show wavy expression patterns, up-regulated at one point but down regulated at another. We performed gene ontology analysis on each of these sets of genes to elucidate the temporal transcriptional program elicited upon LPS stimulation in neutrophils.

All gene ontology annotations were done using the BINGO [31] plugin in cytoscape [32]. Using this algorithm, we assessed for gene set overrepresentation using the hypergeometric test with the multiple correction method of Benjamini & Hochberg False Discovery Rate (FDR) correction. Our gene sets were tested against the background of all predicted human genes. Unless otherwise stated, all biological processes mentioned were significantly (corrected p-value <0.01) overrepresented by the set of genes perturbed at any time after LPS infusion (at t = 0 h). Protein-protein Interaction data were obtained from HPRD, release 9 Protein-protein Interaction data were obtained from HPRD [33].

### Q-PCR for Microarray Validation and in Vitro Neutrophil Gene Expression

From every sample, 250 ng RNA was reverse transcribed to cDNA with superscript III (Invitrogen, Paisley, UK). Gene specific Taqman gene expression assays (Life Technologies, Carlsbad, USA) were used in q-PCR to determine sample gene expression. GAPDH and 18S were used as reference genes. q-PCR was performed at the applied biosystems 7500 fast q-PCR machine using standard procedures.

### In Vitro Neutrophil Stimulation

Neutrophils (5×10^Λ^6 cells/ml) in RPMI supplemented with 0.5% Human Serum Albumin (Sanquin, Amsterdam. The Netherlands) were stimulated at t = 0 with LPS (10 ng/ml; Escherichia coli serotype 055:B5, Sigma Aldrich, purified as described previously [34]), TNFα (10 ng/ml; Abcan, Cambridge, UK), G-CSF (50 ng/ml; R&D Systems, Minneapolis, USA) and GM-CSF (50 ng/ml; Cellgenix, Freiburg, Germany). After 2 hours, 800 µl cell suspension was taken and washed with PBS. Subsequently cells were resuspended in 350 µl RLT with 1% Beta mercapthoethanol and stored at −20°C. After 7 hours, 200 µl cell suspension was used for apoptosis assay. Survival and apoptosis were determined on the FACScalibur by Annexin V apoptosis detection kit (BD, Franklin Lakes, USA) according to the manufacturers’ instructions.

### Statistics

Cell survival was tested with a repeated measures ANOVA, and Tukey’s multiple comparison post-hoc test. P values ≤0.05 were considered statistically significant.

## Supporting Information

Figure S1 **Functional networks of persistent changes in gene expression.** Cohesive network based on 307 downregulated genes. ‘Wavy’ genes are marked red and persistent downregulated genes are represented by large nodes.
(TIF)

Table S1 **List of 2248 perturbed genes on one or more timepoints in alphabetical order.** Fold changes are Log 2 transformed.
(XLS)
