# Supplementary figures and images for: Transcriptome Kinetics of Circulating Neutrophils during Human Experimental Endotoxemia
Source: PLoS One. 2012 Jun 5;7(6):e38255. doi: 10.1371/journal.pone.0038255 (PMC3367952; doi:10.1371/journal.pone.0038255)

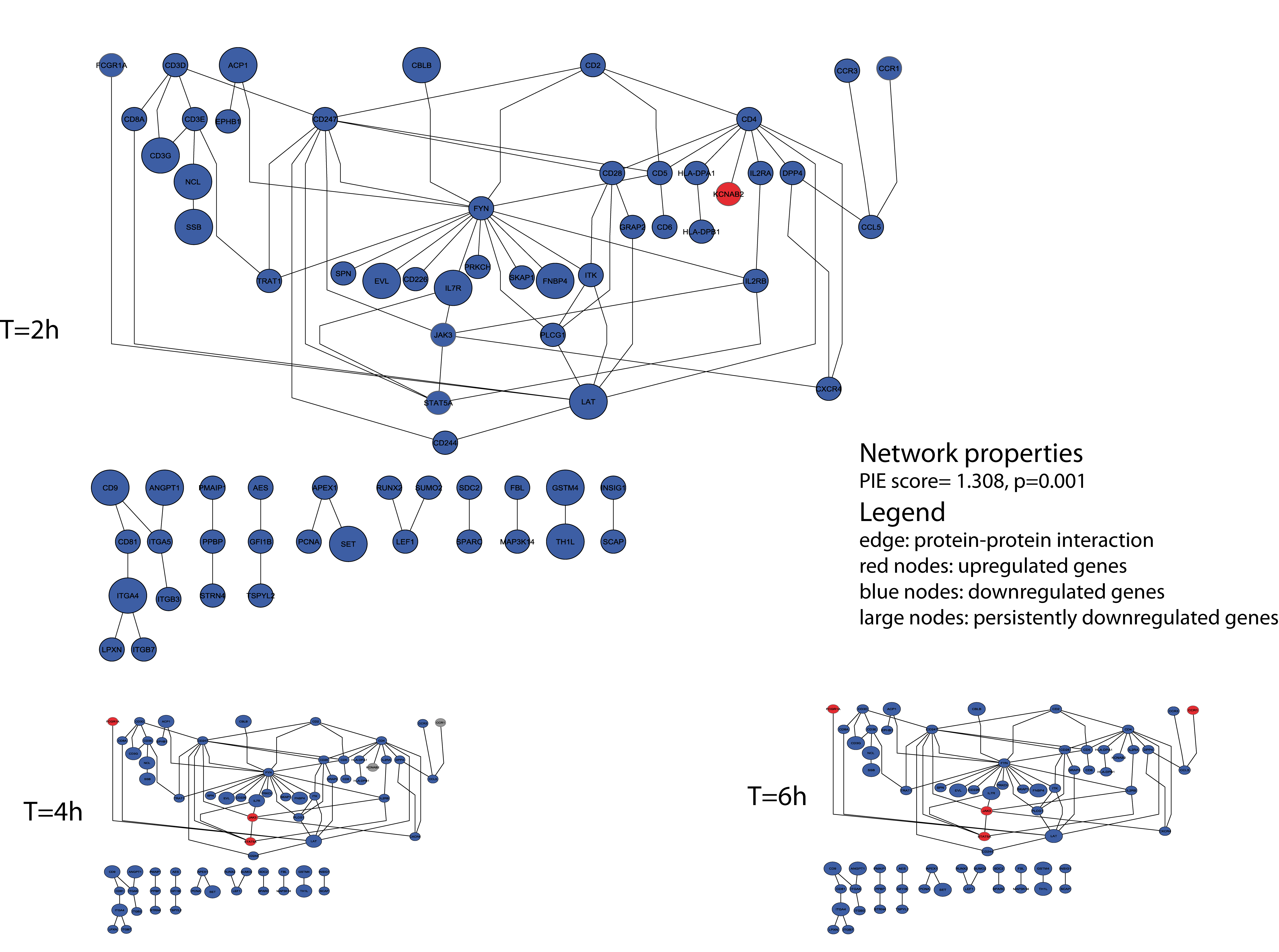

Supplement: Figure S1 — Functional networks of persistent changes in gene expression. Cohesive network based on 307 downregulated genes. ‘Wavy’ genes are marked red and persistent downregulated genes are represented by large nodes. (TIF) [file pone.0038255.s001.tif]
